# Supplementary material for: Determining acute nurse staffing: a hermeneutic review of an evolving science
Source: BMJ Open. 2019 Mar 30;9(3):e025654. doi: 10.1136/bmjopen-2018-025654 (PMC6475222; doi:10.1136/bmjopen-2018-025654)
Supplement: Supplementary data [file bmjopen-2018-025654supp001.pdf]

Supplementary file
Determining acute nurse staffing, a hermeneutic review of an evolving science.

Search Strategy

Database selection
PubMed, CINAHL, arXiv, CiteSeerX, IEEEXplore

Key words/terms (Boolean)
“nursing” arXiv, CiteSeerX, IEEEXplore (as this is a minority term) “nurse staffing” “nurse workload” “hospital staffing” “nursing ratios” “safe staffing” “staffing” AND “patient safety” in PubMed and CINAHL
Years searched 2003-2018

Criteria for inclusion
Primary research, secondary analysis or reviews on any relationship between acute hospital nurse staffing (registered, licenced and unregistered) and an specified outcome.

First iteration used key terms as above.
This yielded 7323 items.
From this 5265 were excluded as these were opinion pieces, editorials, commentaries, proceedings, consensus pieces and papers with no English translation.

2058 Items were initially reviewed
Include systematic reviews (24)
Include primary research (2034)
Exclude other non-peer reviewed work such as reports (23)
Exclude papers which did not meet criteria on reading abstract/full text (1262)
Exclude other material (peer reviewed opinion pieces) (4)
Papers reviewed 769

Mapping and classification by discipline/area of research, major concepts, lens and unit of analysis. The result of this is shown in the main paper.

Critical assessment of the work was for rigour/associations and primarily “gap spotting” To visualise the relationships within discipline areas a Venn diagram was constructed (Figure 2). With the further application for example of Łoś's Theorem, and ultra product could eventually be defined.

Second iteration (as described in methods section)
Search to same criteria adding Nursing AND knowledge intense occupations, safety critical workforce revealed opinion pieces which referenced the terms to describe the labour market but no additional papers reviewed as they did not meet the criteria.
